# Single Channel EEG Artifact Identification Using Two-Dimensional Multi-Resolution Analysis

**DOI:** 10.3390/s17122895

**Published:** 2017-12-13

**Authors:** Mojtaba Taherisadr, Omid Dehzangi, Hossein Parsaei

**Affiliations:** 1 Computer and Information Science Department, University of Michigan-Dearborn, Dearborn, MI 48128, USA; mojtabat@umich.edu; 2 Department of Biomedical Physic and Engineering, Shiraz University of Medical Sciences, Fars Province, Shiraz, Zand, Iran; hparsaee@gmail.com

**Keywords:** electroencephalography (EEG), artifact identification, curvelet transforms, time–frequency representation, multi-resolution analysis

## Abstract

As a diagnostic monitoring approach, electroencephalogram (EEG) signals can be decoded by signal processing methodologies for various health monitoring purposes. However, EEG recordings are contaminated by other interferences, particularly facial and ocular artifacts generated by the user. This is specifically an issue during continuous EEG recording sessions, and is therefore a key step in using EEG signals for either physiological monitoring and diagnosis or brain–computer interface to identify such artifacts from useful EEG components. In this study, we aim to design a new generic framework in order to process and characterize EEG recording as a multi-component and non-stationary signal with the aim of localizing and identifying its component (e.g., artifact). In the proposed method, we gather three complementary algorithms together to enhance the efficiency of the system. Algorithms include time–frequency (TF) analysis and representation, two-dimensional multi-resolution analysis (2D MRA), and feature extraction and classification. Then, a combination of spectro-temporal and geometric features are extracted by combining key instantaneous TF space descriptors, which enables the system to characterize the non-stationarities in the EEG dynamics. We fit a curvelet transform (as a MRA method) to 2D TF representation of EEG segments to decompose the given space to various levels of resolution. Such a decomposition efficiently improves the analysis of the TF spaces with different characteristics (e.g., resolution). Our experimental results demonstrate that the combination of expansion to TF space, analysis using MRA, and extracting a set of suitable features and applying a proper predictive model is effective in enhancing the EEG artifact identification performance. We also compare the performance of the designed system with another common EEG signal processing technique—namely, 1D wavelet transform. Our experimental results reveal that the proposed method outperforms 1D wavelet.

## 1. Introduction

Electroencephalography (EEG) is a method for recording electrical activities of the brain, and is indicated in activities which originate from the cerebral cortex. Examples include brain tumors, encephalopathy, brain death diagnosis, epilepsy, psychiatric disorders, dementia, coma, degenerative diseases related to the nervous system, vertigo, cerebro-vascular disease, and head injury. Detected signals in EEG recordings which have origins other than the brain itself are considered as artifacts. The amplitude of the desired cortical-related EEG component can be somewhat smaller than the amplitude of the artifact signals. Non-cerebral sources of artifacts in the EEG recordings can be as follows: saccade, eye blinking [1,2], respiratory-related artifacts [3,4], electrode placement, cardiac (electrocardiography) artifact [5], broken electrode, and power line noise [6,7,8,9]. Diagnostic information in EEG recordings can be suppressed by these categories of artifacts. Therefore, continuous EEG-based systems demand preprocessing to distinguish between artifacts and target brain activities to annotate noisy segments from clean segments of EEG activities.

User-generated artifacts such as ocular and muscle artifacts are always present in EEG recordings, and almost all EEG recordings suffer from these two categories of noise. There is always a need to preprocess the EEG recording to identify its various components and distinguish them as usable for any further diagnoses. A reliable EEG component identification system enables the practitioner to distinguish between various components presented in the recordings. Based on the literature, any EEG artifact identification system applies the following three major steps to accomplish the identification task:

(1) analyzing the acquired signal in either time or frequency space, or even in frequency and time space jointly.
(2) extracting features in order to characterize various artifacts.
(3) modeling and classification of the artifacts based on the extracted features and assigning specific labels to the contaminated portions of the recorded EEG, such as ocular, muscle, etc.

For extracting features and characterization, various one-dimensional (1D) methods have been proposed [10]. Previous studies have focused on spectral analysis of the EEG signal [11]. These methods include, but are not limited to, parametric models, spike detection of EEG, epoch analysis, methods of clustering, quantitative analysis, and spectral EEG signal analysis. These are based on a quasi-stationarity assumption, require long recordings, and lead to false identification rates because of the presence of various artifacts [12,13,14]. These methods provide spectral information but they do not provide temporal information about a specific event as well.

The other approach to dealing with non-stationary characteristics in EEG is to assume a fundamental non-stationary stochastic model, and then consider the EEG recording as stationary segments. Applications of this category include, but are not limited to, automatic detection of epileptic seizures, neuronal burst discharges, and artifact identification [15]. The difficulties associated with accurate detecting and quantifying these changes, and also efficiently and automatically analyzing them, have restricted the application in the clinical practices.

Other approaches employ frequency and time features jointly [16], or other chaotic features [17,18]. More advanced approaches employ the coefficients of the wavelet transform of EEG recording [2,19], and also a combination of chaotic measures and discrete wavelet transform coefficients [20]. A comprehensive study of EEG artifact identification methods can be found in [6]. Recent artifact identification methodologies include non-linear models [11], artificial neural networks [21], and independent component analysis (ICA) [1].

A specific category of approaches capable of analyzing and detecting non-stationary signals are time–frequency distributions (TFDs). This category allows visualization of the evolution process of the spectral behavior during non-stationary events by mapping a 1D time domain signal into a 2D function of frequency and time. Thus, the TF space enables the extraction of relevant features using approaches such as energy estimation, filter banks, multi-resolution analysis, peak matching, etc. [6]. Because of the non-stationarity in EEG recordings, *instantaneous frequency (IF)*- and TF-based approaches seem to be naturally more proper for EEG artifact identification [22,23].

In this study, we propose a 2D multi-resolution analysis approach to track patterns in TF space, then follow and measure their characteristics. The main goal is to obtain a new feature set capable of enhancing the identification performance. We implement this approach in an identification task and evaluate its performance on a real EEG database. In this study, we apply *2D multi-resolution analysis (MRA)* in order to decompose the given image and then extract discriminative features based on the acquired *sub-bands* for the task of EEG artifact characterization and identification. MRA is as an effective decomposition algorithm that provides a hierarchical view of information which is proper for extracting instantaneous frequency-related features and also geometric patterns in the TF space of a given segment. In this study, we consider various TF analysis representations. Then, we apply 2D MRA analysis using wavelet and its higher-dimensional extension (Curvelet) decompositions. We extract their coefficients and conduct a comparative investigation between our proposed 2D MRA features with a 1D counterpart as the baseline. In this way, we generated and characterized the basis dimensions from the generated feature space using *principal component analysis*. For a comparison between the proposed features and the baseline space, we employed several multi-class classifiers, including (1) linear support vector machine; (2) highly non-linear complex tree; and (3) an instance-based and non-parametric 1-nearest neighbor classifier, for the identification task and then results were reported in parallel. Our experimental results present that the proposed 2D MRA features are capable of characterizing and discriminating between various EEG artifacts with >90% accuracy. Furthermore, as a comparison with the other state-of-the-art studies in single-channel EEG artifact detection, we implement and apply one-dimensional discrete wavelet transformation in the task of EEG artifact detection [24,25,26].

The rest of this study has been organized as follows: The second section presents the EEG data collection procedure and experimental setup, and then provides the necessary background of TF analysis and 2D MRA, and finally describes the procedure of TF analysis and MRA implementation. The third section provides the details of the experimental results over a population of ten participants. Section 4 concludes the obtained results and also provides further insights into the advantages of the proposed framework for EEG artifact characterization and identification.

## 2. Materials and Methods

**Figure 1:**
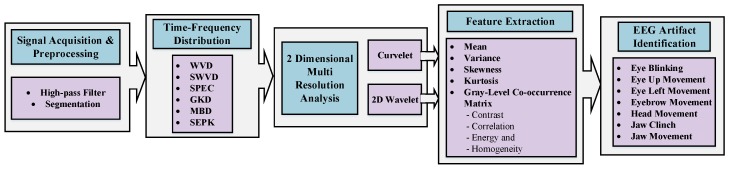
Flowchart of the details of the proposed framework in this study. EEG: electroencephalogram; WVD: Wigner–Ville distribution; SWVD: smoothed-WVD; SPEC: spectrogram; GKD: Gaussian kernel distribution; MBD: modified B-distribution; SPEK: separable kernel.

Figure 1 presents a flowchart of our proposed identification framework. After preprocessing and channel selection, the EEG signal is passed to different time–frequency representation methods. Then, 2D transformations including 2D wavelet and curvelet (as multi-resolution analysis methods) are applied. After extracting features, artifacts are identified and classified into seven classes. In this section, we discuss the implementation of each step in detail.

### 2.1. Experiment Setup and Data Collection

**Figure 2:**
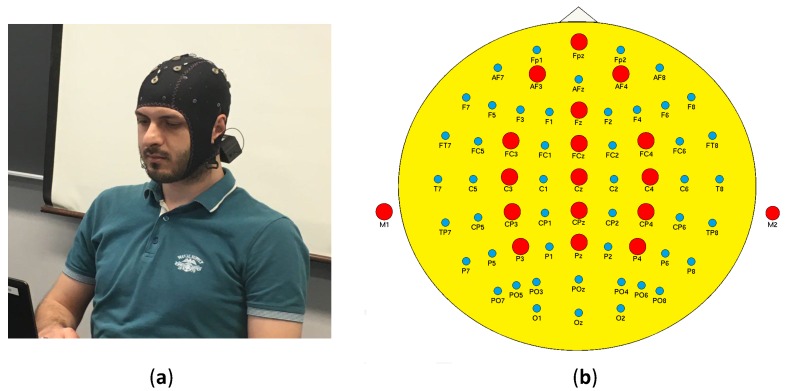
(**a**) Wireless EEG setup attached to a participant to collect data; (**b**) Illustration of channel locations and distribution used in this study.

A 16-channel EEG system with a sampling rate (Fs) of 256 Hz was used for data collection. Right and left earlobes were selected as ground for data acquisition setup, and impedances of the electrodes were clamped less than 5 kΩ. Table 1 shows the parameters of the EEG setup. Electrode locations were chosen to be over the parietal, central, and frontal areas of the skull. Channels’ numbers and names are as follows: Fpz-1, AF3-2, AF4-3, Fz-4, FC3-5, FCz-6, FC4-7, C3-8, Cz-9, C4-10, CP3-11, CPz-12, CP4-13, P3-14, Pz-15 and P4-16. We use these channel names and numbers hereafter. Figure 2 presents the EEG channels’ locations, and also illustrates the data collection setup attached to one of the participants.

We aimed to collect supervised artifact data from ten participants while they were exposed to seven different types of ocular and muscle artifacts, as follows: eye-blinking, eye-up movement, eye-left movement, eyebrow movement, head movement, jaw clinch, and jaw movement. The experiments were conducted at the Wearable Sensing and Signal Processing Laboratory, University of Michigan-Dearborn (Dearborn, MI, USA). The data were collected from ten healthy participants, including six males and four females, all between 20–30 years of age. Neither training nor practice were conducted before the data collection. Participants were instructed to remain stationary while recording in a lab environment. Ten trials of 1-s stationary data were recorded. Additionally, participants were asked to introduce user-generated artifacts such as head and eye movement. One second of each artifact type for ten trials was recorded per participant. Using a beep sound, participants were notified when the recording started and when it ended. Lab Streaming Layer, which is a system of unified collection of time series measurements, was utilized to serve as an interface between the Cognionics data acquisition software and MATLAB. The recorded artifact samples per participant were as follows:

- 1 s of baseline × 10 trials.
- 1 s of eye blinking (no squinting) × 10 trials.
- 1 s of eye movement (up) × 10 trials.
- 1 s of eye movement (left) × 10 trials.
- 1 s of head movement × 10 trials.
- 1 s of eyebrow movement × 10 trials.
- 1 s of jaw clinch × 10 trials.
- 1 s of jaw movement × 10 trials.

Between two trials, there was a 2-s delay to give the participant time to return to the stationary state. The dataset for each participant contains 80 artifact and normal trials. In total, the dataset contains 800 trials of artifacts and normal data.

### 2.2. Preprocessing

EEG recording was first pre-processed using a high-pass filter with cut-off frequency of 0.5 Hz for removing trends. Then, a rectangular window with the length of *L* seconds was used to segment the EEG data. The best size for the *L* is based on the performance of the system (discussed in the Results section). The size of the *j*th segment is equal to L×Fs samples. No overlap was considered between the consecutive segments. The facial-related signals will be predominant in the frontal regions of the brain. Therefore, we only considered channel Fpz as the representative of the other channels, as it is the closest channel to facial muscles, which are in turn the source of the artifacts considered in this study. Furthermore, previous research studies on ocular and facial muscle artifacts revealed that Fpz is the most prone channel to be affected by these categories of artifacts [21,27,28]. Based on this assumption and hereafter, we investigate Fpz for our proposed method of single channel two-dimensional multi-resolution analysis. This analysis can be extended to multi-channel-based identification systems based on the discriminative ranking of the EEG channels.

### 2.3. Time–Frequency Analysis and Representation

Time–frequency analysis could be considered as non-stationary signals analysis with frequency content varying with time. Time–frequency distribution (TFD) is a suitable representation for non-stationary and multi-component signals which is able to describe the energy distribution of the given signal over time and frequency space simultaneously. The TFD presents the beginning and end times of the different components of the signal as well as their frequency scope. TFD is also capable of presenting variation in both time and frequency, namely instantaneousfrequency(IF) [29]. IF introduction requires an explanation of the analytic signal. For signal x(n) with real values, we correlate a complex value signal $x_{h}\left(n\right)$ which is defined as

(1) $$x_{h}\left(n\right)=x\left(n\right)+iHT\left(x\left(n\right)\right)$$

where HT(x(n)) is the Hilbert transformation of the input signal x(n). $x_{h}\left(n\right)$ is the analytic version of the x(n). The interpretation of this definition in the frequency space is simple since $x_{h}$ is a Fourier transform in which the negative frequency component has been eliminated, the positive values have been doubled, and the DC part is kept unchanged:

(2) $$\begin{array}{cc} F_{h}\left(w\right)=0 & \;\;\;if\;\;w<0\\ F_{h}\left(w\right)=F\left(0\right) & \;\;\;if\;\;w=0\\ F_{h}\left(w\right)=2F\left(w\right) & \;\;\;if\;\;w>0 \end{array}$$

where *F* is the Fourier transform of the *x*, and $F_{h}$ is the Fourier transform of $x_{h}$. Therefore, it is possible to acquire an analytic signal from a real signal by multiplying its spectrum by zero for the negative frequencies, which does not change the information content due to the fact that for a real signal, $F\left(-w\right)=F^{*}\left(w\right)$. The resulting output can be used to define the concepts of IF and instantaneous amplitude (IA) in a unique way by:

(3) $$\begin{array}{cc} f\left(n\right)=\frac{1}{2\pi }\frac{d\;argx_{h}\left(n\right)}{dn} & \;\;\;instantaneous\;frequency, \end{array}$$

(4) $$\begin{array}{cc} h\left(n\right)=|F_{h}\left(w\right)| & \;\;\;instantaneous\;amplitude \end{array}$$

The notion of IF assumes that at each time *n*, there should be only one single frequency component. The single IF concept is not applicable to multi-component signals. To apply IF law to multi-component signals, it can be assigned to every component of the signal separately. Different IF estimation methods for multi-component signal analysis have been considered in the literature [29]. These methods extract and localize signal components from TF representation of the signal. Then, they implement an IF estimation procedure [30]. Any typical implementation of IF estimation for a multi-component signal includes two major steps:

(a) Transformation to TF space—first, the given signal is mapped to the TF domain using a suitable TFD approach.

(b) Detecting local peaks in TFD and linking the components—the output of TFD (R(T,F)) is considered as a representation in 2D space of frequency and time by the IF estimation approach. Frequency and time are considered as the column and row of the 2D space, and by applying a derivative test (first and second) they characterize local extremums. Extremums with values lower or higher than a specific threshold are counted as valid peaks. Then, an algorithm designed to detect connected components by evaluating the connectivities in pixels and counting the number of connected pixels, detects the connected components in R(T,F). TFD selection for EEG signal representation is the first step in identification and detection system designation in the TF space. A proper TFD highlights the non-stationarities in the input signal that enables the system to discriminate between considered classes. To generate an efficient feature set, a TFD approach should satisfy the following constraints:

- ***IF Rule*:** Peaks in TFD space of the signal under consideration should be capable of estimating the IF of the signal.
- ***Real Values*:** R(T,F) must have real values.
- ***High Resolution*:** The reduced interference property is achieved while preserving a good TF resolution.
- ***Reduced Interference*:** The TFD attenuates the unwanted cross-terms in the TF domain relative to the signal terms.
- ***Energy*:** The energy of a specific portion of the input signal can be considered as a simple integration of TFDs over a related area.

Two-dimensional representation techniques are categorized as a group of techniques that process the TF distribution of a recorded signal. Many studies have applied a variety of approaches to the TFD to select a proper methodology for their application, helping to improve the resolution, robustness, precision, or performance. Based on the previous studies, the suitability of the TFD approach is data- and application-oriented [31].

A review of the recent methods for TF representation reveals that they can be categorized in six groups as follows: Gaussian kernel, Wigner–Ville distribution (WVD), spectrogram, modified-B, smoothed-WVD, and separable kernel. Reduced interference approaches such as Smoothed-WVD are capable of improving the quality of the representation. This is because decreasing the interference results in a reduction in the effect of cross-terms [32].

Our aim is to assess the mentioned approaches to determine their performance regarding our specific application in this study (i.e., artifact identification). Table 2 lists the kernels for the TFDs used in this paper. The author in [22] provides a gentle and comprehensive introduction to this area.

**Figure 3:**
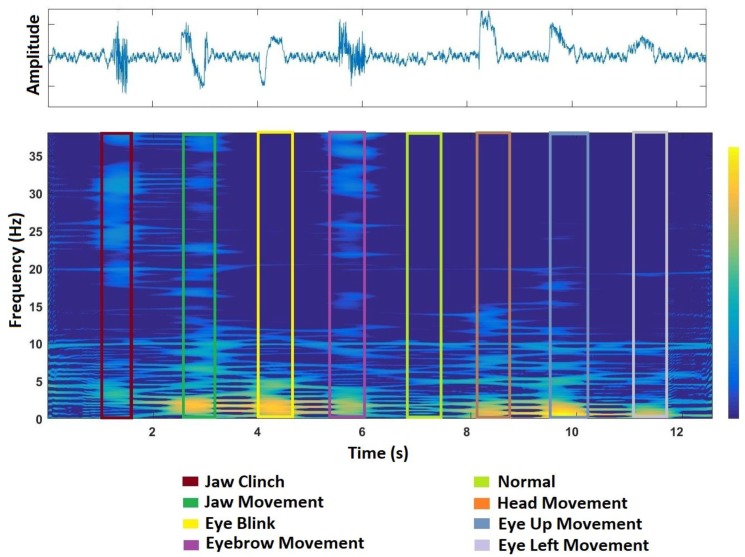
EEG signal representation. (**top**): Time domain; (**bottom**): TF domain using the SWVD approach.

Figure 3 depicts the TF representation of a sample set of artifacts using the SWVD approach. This is generated by concatenating seven artifact segments and a normal segment of EEG from the Fpz channel. Figure 3 illustrates the SWVD representation of different categories of artifacts highlighted in different colors.

### 2.4. Multi-Resolution Analysis (X-Let Transforms)

Multi-resolution analysis (MRA) was introduced to EEG signal characterization by Clark [33]. MRA decomposes the input data to different levels to characterize their patterns and intensities in the input data. MRA application for EEG artifact identification is based on the idea that many of the ocular and muscle artifacts may have specific characteristics in time and/or frequency domains (Figure 3). They can distort the EEG only in a limited time or frequency range. Characteristics of the artifact result in some specific IFs and patterns in time–frequency (TF) space which can be analyzed with MRA decomposition methods. In this study, we consider X-let transformations (2D wavelet transform and curvelet transform) as 2D MRA methods for the purpose of artifact identification from TF space.

#### 2.4.1. Two-Dimensional Wavelet

The basic concept of wavelet transforms is to provide multi-scale analysis, which means taking apart and analyzing a function in more detail. Two-dimensional (2D) wavelet is a natural extension of the single dimension case. To use the wavelet transform in two dimensions, first a 1D filter bank must be applied to the rows of the TF space, and then applied to the columns. If the given image has $M_{1}$ columns and $M_{2}$ rows, applying a one-dimensional (1D) filter bank to the rows results in two sub-band images; each sub-band image has $M_{1}/2$ columns and $M_{2}$ rows. Then, applying 1D filter bank to the columns of the two resulting sub-band images results in four new sub-band images; each sub-band image has $M_{1}/2$ columns and $M_{2}/2$ rows. The 2D filter bank concatenates four sub-band images to generate the given input TF space of size $M_{1}$ by $M_{2}$ [34].

**Figure 4:**
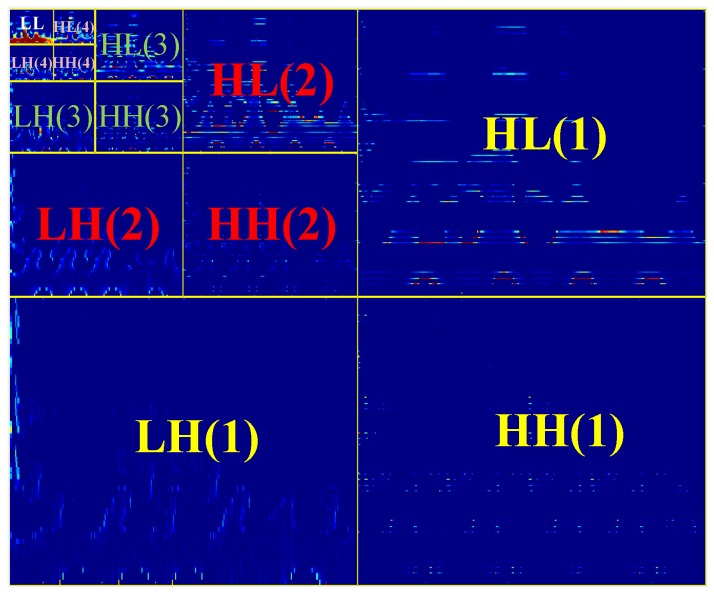
Structure of four-level wavelet transformation with *Haar* mother wavelet applied to one segment of the EEG signal. *k* in HH(k), HL(k), LH(k) and LL(k) indicates the *k*th level.

*N* levels wavelet decomposition results in 3N+1 matrices of coefficients. One approximation and 3×N details matrices, *N* diagonal, *N* vertical, and *N* horizontal matrices for each level. Each sub-band carries specific information about the input image. Figure 4 illustrates the sub-bands of four-level 2D wavelet transformation applied to one segment of EEG data. According to Figure 4:

LLquadrant: The upper-left portion presents all of the coefficients of the wavelet decomposition, which resulted from applying a low-pass filter HL to the given TF image. This block is an approximation of the input image, and is called LL in this study.

HHquadrant: The lower-right portion is similar to the upper-left one except the high-pass filter HG is applied for coefficient extraction. This portion contains overall patterns from the given image in the diagonal direction.

HL\LHquadrant: The lower-left and the upper-right portions are filtered using HL and HG, respectively. HL and LH portions contain vertical and horizontal patterns from the given image, respectively.

In this study, 2D wavelet decomposition with *Haar* mother wavelet at four levels was applied to smoothed Wigner–Ville distribution time–frequency representations. *Haar* mother wavelet has mainly been used for signal and image processing, since it is computationally efficient. With four levels of wavelet decomposition, we have 13 matrices of coefficients (12 matrices for details and one matrix for approximation). For each matrix of coefficients, we extracted the following features: variance, kurtosis and skewness, mean, and second-order statistical features extracted from a gray-level cooccurrence matrix. The features extracted from the cooccurrence matrix include contrast, correlation, energy, and homogeneity (based on Table 3). Figure 4 illustrates the representation of four levels 2D wavelet transformation applied to one segment of EEG TF representation. Each level represents the data from different orientations (vertical, horizontal, and diagonal).

Wavelets succeed in providing a sparse framework for the signals. However, this efficient representation is restricted to 1D signals because of the nature of this transform. Sparse structures and linear and nonlinear trends in 2D space (e.g., lines and curves) could have a sparse representation. Wavelets may fail to provide such a sparse representation, since obtaining 2D wavelets by only three directions (vertical, diagonal, and horizontal) may not be able to exploit local correlatedness within 2D signals. Therefore, wavelet’s limited directions raises the need for the next generation of *multi-directional 2D multi-resolution analysis*.

#### 2.4.2. Curvelet

**Figure 5:**
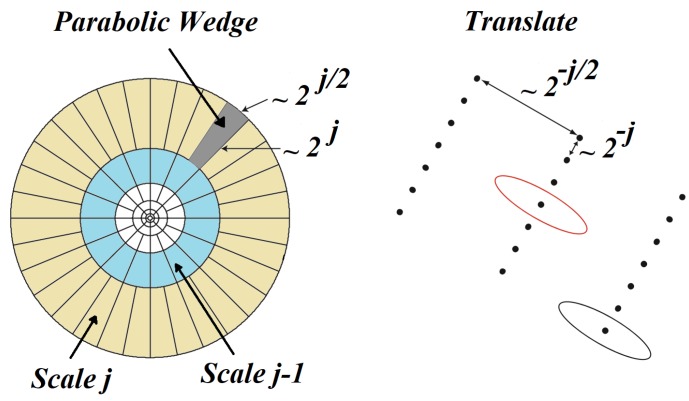
Curvelets parameterized by scale, location, and orientation. *j* represents the *j*th scale.

A new member of the family of multi-resolution analysis is curvelet. It is the next, higher-dimensional, version of the wavelet transformation designed with the aim of decomposing 2D data in different angles and scales. The idea behind curvelet transformation is to represent a curve shape as a superposition of other functions with different widths and lengths which obey the law of parabolic scaling $\left(length^{2}=width\right)$. Using this scaling, decreasing the scale as a power law results in an increment in anisotropy. Applying a dyadic decomposition to the frequency space doubles the length of each localizing window at each of the other dyadic sub-bands. Figure 5 represents the curvelet scales and curvelets parameterized by scale, location, and orientation.

**Figure 6:**
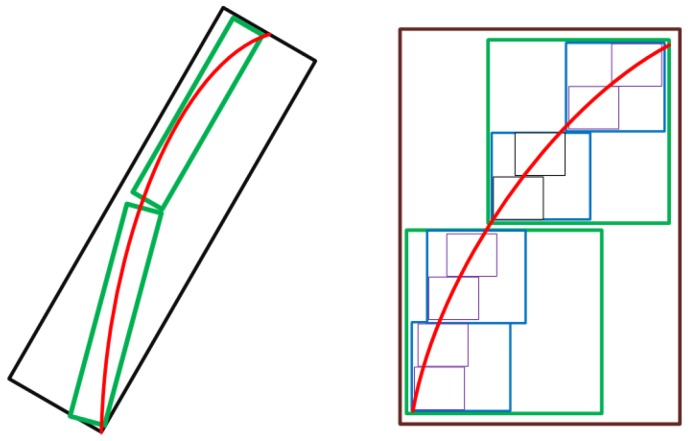
Edge representation and matching by wavelet and curvelet. A lower number of curvelets decompose an edge more efficiently in comparison with wavelets. (**left**): Curvelet; (**right**): Wavelet.

This feature gives curvelets the ability to represent the signature patterns in the TF space much more efficiently by capturing singularities along curves of the patterns, and results in better IF estimates. Figure 6 shows edge representation and matching by wavelet and curvelet transforms and how the curvelet transform is more efficient in comparison with wavelet. Curvelet is capable of using a lower number of coefficients to represent a smooth contour while keeping the precision.

- Curvelet Implementation:

To define a curvelet transform, suppose that we work in the space $R^{2}$ with a given 2D input P[k,l] with dimensions *K* and *L*; the discrete curvelet transformation, $DCT^{D}\left(c,d,\beta \right)$, is achieved as

(5) $$DCT^{D}\left(c,d,\beta \right)=\sum_{0\leq k<K}^{}\sum_{0\leq l<L}^{}P\left[k,l\right]\Psi_{c,d,\beta }^{D}\left[k,l\right]$$

Equation (5) has been implemented in frequency space. It can be written as follows:

(6) $$DCT^{D}\left(c,d,\beta \right)=IFFT\left(FFT\left(P\left[k,l\right]\right)\times FFT\left(\Psi_{c,d,\beta }^{D}\left[k,l\right]\right)\right)$$

where $DCT^{D}$, $\Psi^{D}$, *c*, *d*, and β are coefficients of the discrete curvelet transform, curvelet function, scaling parameter, location parameter, and orientation parameter, respectively. A detailed explanation of the implementation of Equations (5) and (6) can be found in [35].

Curvelet implementation approaches can be grouped into two classes. The first class is based on the wrapping of specially selected Fourier samples, while the other is based on an unequally-spaced fast Fourier transform (USFFT).

The difference in these two approaches is just in choosing the spatial grid which is used in translating curvelet at each angle and scale. Both approaches return a curvelet coefficient based on the orientation, spatial location, and scale parameters. USFFT irregularly samples the Fourier coefficients of the input *f* to obtain the curvelet coefficients. However, the wrapping approach finds the curvelet coefficients using a wraparound technique and various translations. The output for both approaches is the same, but the wrapper is computationally more efficient. Therefore, curvelet via wrapping is used in this study.

**Figure 7:**
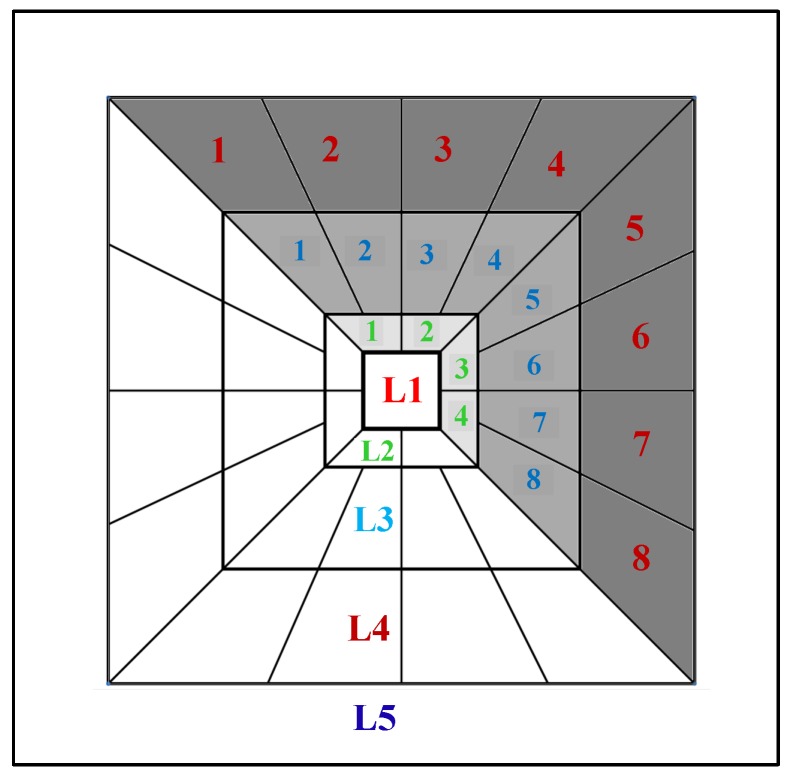
Representation of five levels of curvelet transform, and considered scales in this study. L1, L5 and gray scales (half of the scales L2, L3 and L4) are the selected ones in this study.

- Curvelet Coefficient Matrix Selection and Feature Extraction:

In order to extract features from time–frequency (TF) input $f_{i}\left[m;n\right]$, it was decomposed using the discrete curvelet transformation via the wrapper approach. Five scales of decomposition were used. Five decomposition bands are as follows: (1) Scale one as approximation; (2) Scale two as details; (3) Scale three as details; (4) Scale four as details; and (5) Scale five as finest details. The number of angles at the second level of decomposition was set to eight [36]. The first matrix is coarse scale and includes the low-frequency component of the image. The next scales contain the high-frequency component of the TF input $f_{i}$. Based on the orientation, scales 1 to 5 have 1, 8, 16, 16, and 1 matrices of coefficients, respectively. Since a curvelet with angle θ generates the same coefficient as the other curvelet at angle θ+π, then we only use half of the sub-bands at scales 2–4. Figure 7 presents the selected matrices in this study. In Figure 7, Li means level *i*, and numbers 1, 2, 3, etc., are the orientation numbers in levels 2–4. We selected these sub-bands in this study. Therefore, a total of 22(=L1(1matrix)+L2(4matrix)+L3(8matrix)+L4(8matrix)+L5(1matrix)) matrices of curvelet coefficients were selected.

**Figure 8:**
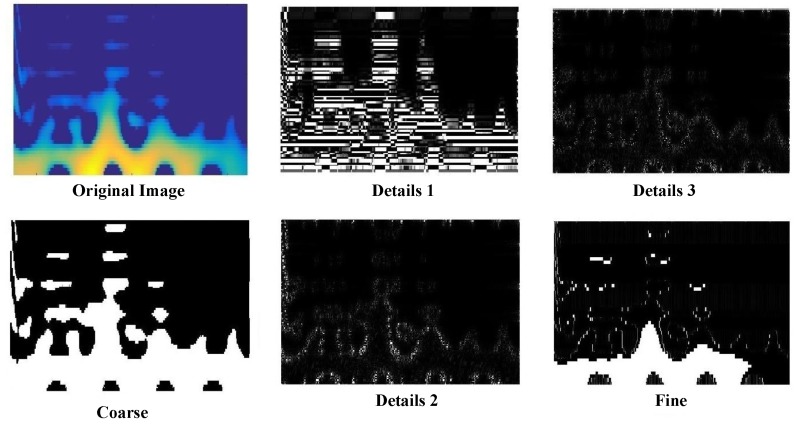
The result of curvelet transformation applied to one segment of EEG TF representation. Curvelets here were manually thresholded for visual observation.

The extracted geometrical and statistical features from curvelet coefficient matrices are as follows: a co-occurrence matrix and its related properties including correlation, entropy, contrast, and homogeneity (based on Table 3). Furthermore, first- and second-order statistical features including third- and fourth-order moments (kurtosis and skewness), variance, and mean have been extracted. We have added kurtosis and skewness of curvelet coefficients at a given scale as texture features, since together they provide more discriminative capability as the TF patterns’ boundaries are implicitly captured by curvelet coefficients. Thus, eight features were extracted for each matrix. A feature vector with 176 columns (8×22=176) was generated for each TF input $f_{i}$. Figure 8 shows the result of curvelet transform at scale 5 for one segment of EEG TF representation.

### 2.5. Predictive Model

Complex decision tree is a data-driven rule-based predictive method which attempts to employ as many features as possible in order to expand the decision tree and fit the training data. The resulting rule base is interpretable by humans and can be used for knowledge acquisition. We used information gain as the splitting criterion ([37], Section 6.2).

Support vector machine (SVM) is a discriminative robust linear classifier that aims to maximize the margin between different classes of data using a quadratic objective function. We employed soft margin SVM with a Gaussian kernel in order to add the nonlinear capability of the resulting hyperplane [38].

K-nearest neighbor (K-NN) (*K* = 1 in this study) is a non-parametric distance-based classifier which is highly non-linear and employs the training data themselves to evaluate and label unseen data. The choice of predictive methods was made based on different and complementary properties among them [39]. Using the above-mentioned different classifiers, we aimed to identify the characteristics of the extracted feature sets.

## 3. Results and Analysis

In this section, we evaluate the performance of the proposed EEG artifact identification scheme using time–frequency (TF) analysis and multi-resolution analysis (MRA). The data set we used has been described in Section 2.1 and Section 2.2. For each participant, the data consists of one channel EEG signal which includes seven artifact trials and one normal session. Features were extracted from the TF representation of each segment of the EEG data with the length of T seconds based on Section 2.4.1. TFDs discussed in Section 2.3 have been considered to generate TF images for EEG segment, including Wigner–Ville distribution (WVD), spectrogram (SPEC), modified B-distribution (MBD), Gaussian kernel distribution (GKD), and Smoothed WVD (SWVD). The parameters for GKD and MBD have been chosen as α=0.8 and β=0.02, respectively. These values have been selected based on the previous research studies and investigations on theoretical and practical applications of TF representation of EEG signal using GKD and MBD approaches ([22], Sections 7.4 and 15.5), [40,41]. A *Hanning* window has been chosen for SPEC and SWVD, with a length of [Fs/4] samples.

Ten-fold cross-validation (10-CV) multi-class SVM, complex decision tree, and 1-nearest neighbor (1-NN) trained with the extracted feature sets have been selected for the evaluation procedure. In 10-CV, the original dataset is partitioned into 10 equal-size subsets. Of the 10 subsets, a single subset is retained as the validation data for testing the model, and the remaining nine subsamples are used as training data. The cross-validation process is then repeated 10 times (the folds), with each of the 10 subsets used exactly once as the validation data. The 10 results from the folds can then be averaged to produce a single estimation. The advantage of this method is that all observations are used for both training and validation, and each observation is used for validation exactly once.

### 3.1. Artifact Identification Result Comparison

**Figure 9:**
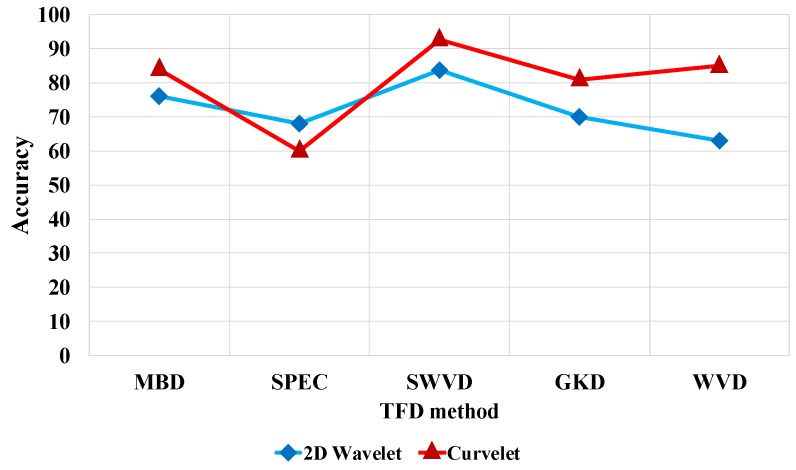
Accuracy for 2D wavelet and curvelet transformations versus TFDs using support vector machine (SVM) classifier and one-second window length.

**Figure 10:**
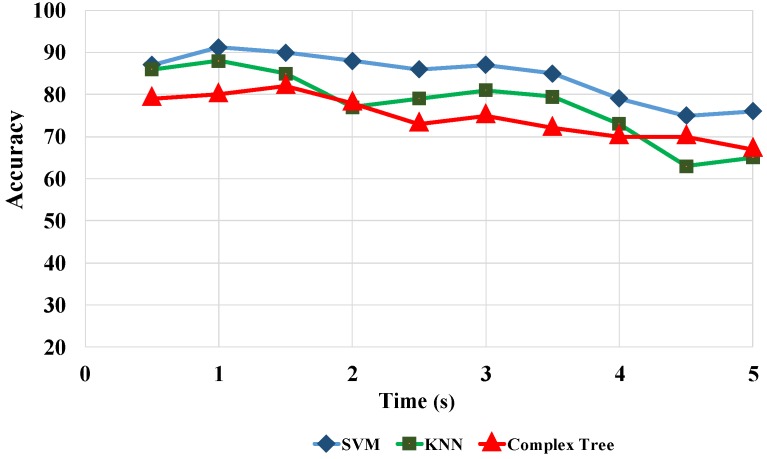
Accuracy versus time window length for SVM, complex tree, and 1-nearest neighbor (1-NN) classifiers.

In this section, we compare the accuracies obtained by different TFDs, classifiers, time window lengths, and 2D MRA methods to identify seven different artifact classes using various performance measures. Figure 9 illustrates the comparison of the results for five TFDs and two X-let transformations. Figure 9 shows that SWVD outperforms the rest of the TFDs when associated with curvelet transformation. A comparison between three classifiers and time intervals based on curvelet transformation and SWVD in Figure 10 reveals that the SVM predictive model and 1-s window achieve the best results. As Figure 9 and Figure 10 present, the window length of 1 s, SWVD TF method, and SVM predictive model demonstrated the best performances in our experiments.

#### 3.1.1. Population Average Identification Result Comparison

Table 4 presents the results of the SVM model for different classes of artifacts using a 1-s time interval and the SWVD TF method. Statistical parameters of the SVM classifier including total accuracy, specificity, and sensitivity have been provided in Table 4. The table also reports a comprehensive comparison between the average performance results over all participants using our proposed 2D MRA features using (1) 2D wavelet; (2) curvelet transforms; and (3) 1D discrete wavelet transform (DWT), which is a state-of-the-art method for EEG artifact identification [42,43,44,45,46]. In order to obtain the EEG artifact identification results using 1D DWT, we extracted a set of features from the sub-bands of DWT, and support vector machine (SVM) as a predictive model. The wavelet transformation gives us the multi-resolution description of a non-stationary signal. Low- and high-pass filters are repeatedly applied to the signal, followed by decimation by 2, to produce the sub-band tree decomposition to some desired level. The low- and high-pass filters are generated using orthogonal basis functions. The length of these filters is chosen as twice the length of the signal. The DWT consists of $log_{2}\left(N\right)$ levels at most. At each decomposition level, two sets of coefficients, approximations, and details are generated. DWT of level five was applied to the EEG recordings to reach the approximate frequency ranges of the α, β, δ, and θ sub-bands. After decomposing the signal in each window, the following features were extracted from the sub-bands:

- Average power of the coefficients.
- Mean of the coefficients.
- Standard deviation of the coefficients [47].

**Figure 11:**
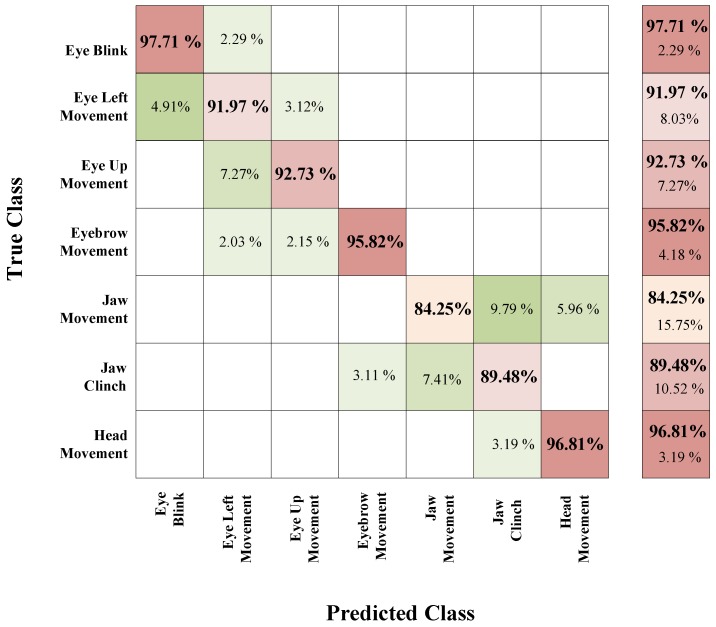
Confusion matrix for participant 1, SVM predictive model, 1-s window length, and SWVD TFD.

Figure 11 shows the confusion matrices for the SVM predictive model, EEG segments of length T=1 s, SWVD method, and curvelet transform. For a specific artifact, each row of the figure presents the overall percent of segments which were correctly identified, and also the percent of the segments confused with the other classes. In each class, the total number of segments is 70. The results show that jaw clinch is confused with eyebrow movement and jaw movement 3.11% and 7.41% of the time, respectively; likewise, jaw movement is the first contributor to the total error, which is confused with head movement and jaw clinch 5.96% and 9.79% of the time, respectively.

In order to compare the 2D wavelet and curvelet results, we conducted a statistical test with the null hypothesis that the performance accuracies of the 2D wavelet and curvelet on different participants comes from independent random samples from normal distributions with equal means, using a two-sample *t*-test. The alternative hypothesis is that the 2D wavelet and curvelet performance accuracies come from populations with unequal means. The test rejected the null hypothesis at the 0.5% significance level.

#### 3.1.2. Statistical Analysis and Comparison of the Identification Results

Table 5 investigates whether the accuracy improvements via the curvelet method are statistically significant. To do so, a one-tailed Wilcoxon signed-rank test [48] was employed to compare the results achieved by “curvelet” compared to “2D wavelet” and “1D wavelet”. The confidence intervals of the differences in the results with respect to the curvelet/wavelet methods are shown in Table 5. Positive confidence levels imply that the curvelet method had a better performance than the comparative method, while negative confidence levels indicate otherwise. Confidence levels higher than 90% are commonly considered as significant improvements in the results. It can be observed in Table 5 that the curvelet method significantly improved the results achieved by the 1D wavelet and 2D wavelet methods. This further verifies the effectiveness of the proposed high-dimensional multi-resolution analysis method (curvelet). Table 5 also shows that the 1D wavelet method achieved better results compared to the 2D wavelet method. However, the differences in the results are not statistically significant.

#### 3.1.3. Participant-Specific Identification Accuracy Comparison

**Figure 12:**
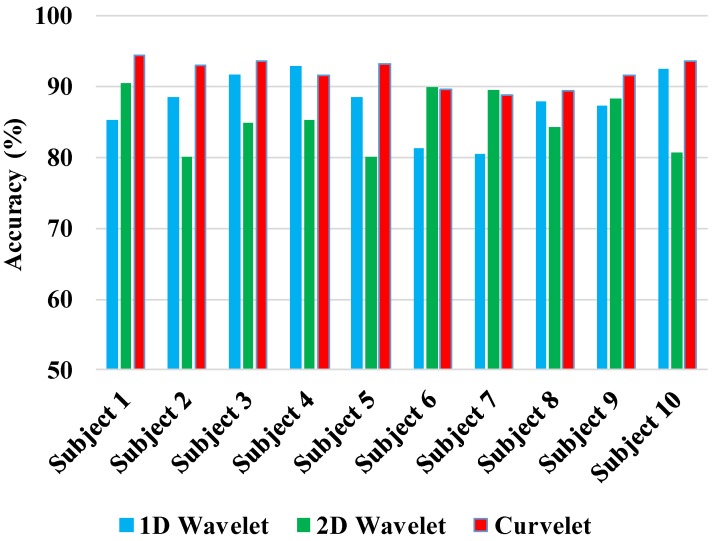
Total accuracy for 10 participants.

Figure 12 reports the results for 10 different participants. Results are based on the 10-CV SVM classifier, 1-s window length, and SWVD TFD. As Figure 12 presents, curvelet transform had the best performance in comparison with 1D wavelet and 2D wavelet transformations consistently over all participants. Comparing the results of 1D vs. 2D wavelet analyses, there is no dominant trend, and depending the participant, one led to better results than the other. The observations in Figure 12 suggest that participant-specific results are generally in line with the population average results.

### 3.2. Principal Component Analysis (PCA) Results

Due to the high dimensionality of the curvelet feature space, the curse of dimensionality might negatively affect the generalization performance. Feature transformation techniques aim to reduce the dimensionality in the data by transforming data into new spaces. The intuition behind most of these approaches is that the useful portion of data lies near the low-dimensional manifold that is a part of the original high-dimensional space. Recent approaches have tried to efficiently identify these manifolds and then extract them. In this study, we applied PCA [49] with the aim of reducing the dimensionality of the feature set.

**Figure 13:**
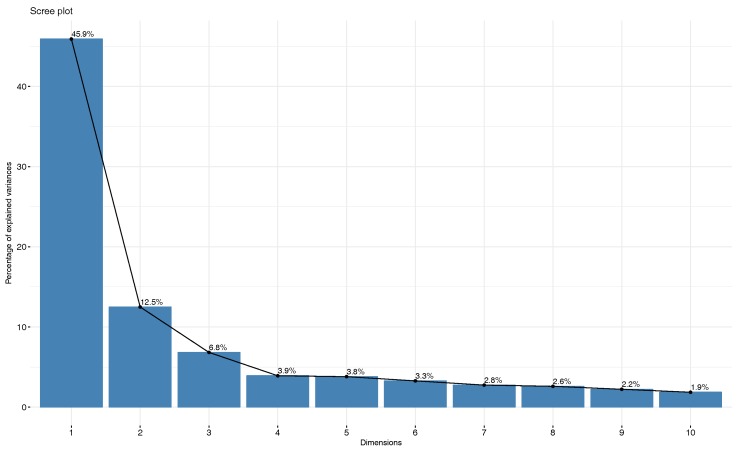
Resultant principal components (PCs) by decomposing the data segments.

We employed PCA to the features extracted from segments and analyzed the resulting principal components (PCs) in order to detect the most descriptive bases of artifacts data. Since the PC space is orthonormal, we can simply remove the dimensions without affecting others. Figure 13 illustrates the resultant PCs by decomposing data segments recorded from participant 1 using PCA transform.

**Figure 14:**
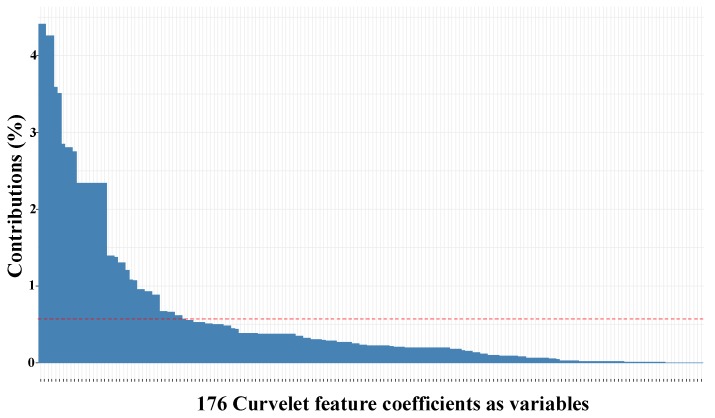
Contribution of variables to PC1.

As shown in Figure 13, most of the contribution to the variance of the data (>85%) was summarized in the first ten principal components (PCs). Therefore, we kept the first ten components of the data for the subsequent predictive model training. In order to characterize the PCs, we also generated the contribution of the curvelet coefficients as variables to each of the PCs. Figure 14 demonstrates the contribution of curvelet variables to PC#1. As shown in Figure 14, the contribution is shared between many of the curvelet coefficients. Based on our observation, variables from different ranges of coarse to fine representations of curvelet contribute to the PCs. We noticed that coefficients from fine representations contributed more than the coarse representations, which might be the result of capturing diagonal and nonlinear trends in the data by curvelet transform, as shown in Figure 8. Similar observations were made with other participant data, such that there was always a combination of the curvelet coefficients from different levels that contributed and described the PCs as the bases of the curvelet space.

#### Identification Result Comparison after Applying PCA

Table 6 reports the results of applying PCA on ten participants’ data. Applying PCA improved the average accuracy of 2D wavelet and curvelet by 3.27% and 1.11% over the population of participants, respectively. The relative improvement of PCA transformation on wavelet space was 20.07%. The relative improvement of PCA transform on curvelet was 15.28%. The improvement caused by PCA is likely to be due to the fact that the original high-dimensional wavelet and curvelet spaces are summarized into a reduced basis vector space that are more aligned with the distribution of data (i.e., principal vectors). In this way, the redundancy in the high-dimensional space is eliminated, and as a result, the generalization ability of the predictive model is improved.

## 4. Discussion

In this study, we considered the possibility of improving the identification and localization of EEG artifacts by proposing a generic approach that combines three complementary techniques, including TF analysis, multi-resolution analysis, and machine learning. In this study, we investigated different approaches for the TF representation of EEG segments, as well as a new approach (MRA) to decompose the TF images in order to capture information in different resolutions and image sizes. In particular, in this study, we implemented a MRA algorithm on the TF spaces using wavelet and curvelet decomposition methods in order to identify and localize EEG artifacts. The intuition behind using decomposition (multiple resolutions) is that a feature in a TF image may lie on the first resolution and the other feature may lie on the next resolution. An image could have features with low contrast and small size, and the other image could have features with high contrast and large size. In real applications, a sample image contains both types of features. Therefore, decomposition provides an analysis of all the features lying on different levels of resolution. An empirical comparison of the proposed method with another common EEG signal processing method (1D wavelet) demonstrated that the 2D MRA approach outperformed it when used for EEG artifact identification.

## Figures and Tables

**Table 1:** Parameters of EEG setup.

Parameter	Description
Amplifier	16-channel device (Cognionics, Inc., San Diego, CA, USA)
Sampling frequency	256 Hz
High-pass filter	0.5 Hz
Electrode arrangement	16-channel subset of 64-channel 10–20 systems (presented in Figure 2)
Ground reference	Left and Right earlobes
Electrode type	Ag/AgCl
Recording platform	Our exclusive software

**Table 2:** Six different time–frequency distributions (TFDs) used in this study and their related kernels. γ is a real positive value and W[l] shows the basic function for a window. $Hann_{l}\left[l\right]$ and $Hamm_{m}\left[l\right]$ are Hanning and Hamming functions, respectively.

TFD	Kernel	Kernel Type
SEPK	$Hamm_{n}\left[l\right]Hann_{l}\left[l\right]$	Separable kernel
SWVD	δ[l]W[n]	Lag-independent Kernel
GKD	$\frac{\surd \pi \sigma }{2|m|}e^{\frac{-\pi^{2}\sigma l^{2}}{4n^{2}}}$	Product kernel
MBD	$\frac{cosh^{-2\gamma }}{\sum_{l}^{}cosh^{-2\gamma }l}$	Lag-independent
SPEC	W[l+n]W[l−n]	Non-separable kernel
WVD	δ[l]	Lag-independent Kernel

**Table 3:** List of statistical features extracted from the gray-level co-occurance matrix (GLCM).

Property	Description	Formula
**Contrast**	provides the difference between a pixel and a neighbor over the given matrix	$\sum_{k,l}^{}{\left|k-l\right|}^{2}p\left(k,l\right)$
**Correlation**	provides correlation value between a pixel and a neighbor over the given matrix	$\sum_{k,l}^{}\frac{\left(k-\mu k)(l-\mu l)p(k,l\right)}{\sigma_{k}\sigma_{l}}$
**Energy**	provides the squared summation of the elements of the given matrix	$\sum_{k,l}^{}p{\left(k,l\right)}^{2}$
**Homogeneity**	provides the closeness of the distribution of elements in the given matrix	$\sum_{k,l}^{}\frac{p(k,l)}{1+|k-l|}$

**Table 4:** Achieved accuracies for each artifact of all participants, 1-s window length, SWVD TFD, and SVM predictive model.

	Sensitivity			Specificity			Total Accuracy		
	1D Wavelet (%)	2D Wavelet (%)	Curvelet (%)	1D Wavelet (%)	2D Wavelet (%)	Curvelet (%)	1D Wavelet (%)	2D Wavelet (%)	Curvelet (%)
**Eye blinking**	96.02	92.15	97.91	99.58	99.02	99.72			
**Eye left movement**	91.05	81.24	91.97	99.02	97.91	99.16			
**Eye up movement**	85.53	85.45	92.73	98.33	98.32	99.15			
**Eyebrow movement**	89.43	83.49	95.82	98.75	98.19	99.51	89.57	83.71	92.71
**Jaw movement**	83.84	79.78	84.25	99.31	97.77	98.19			
**Jaw clinch**	88.43	76.68	89.48	98.73	97.36	98.87			
**Head movement**	92.75	87.12	96.81	99.45	98.61	99.59			
**Average**	**89.57**	**83.71**	**92.71**	**99.02**	**98.17**	**99.17**	**89.57**	**83.71**	**92.71**

**Table 5:** Statistical test of the results achieved by the proposed 1D wavelet and 2D wavelet in comparison with the curvelet method.

System	2D Wavelet	Curvelet
**1D Wavelet**	−89.74%	91.65%
**2D Wavelet**	NA	96.47%

**Table 6:** Accuracies for 10 participants before and after applying principal components analysis (PCA) (%).

	Total Accuracy			
	2D Wavelet		Curvelet	
**Method**	**-**	**PCA**	**-**	**PCA**
**participant 1**	81.71	84.34	93.71	94.31
**participant 2**	79.03	83.51	94.91	96.15
**participant 3**	80.01	87.56	95.61	97.32
**participant 4**	83.26	87.21	92.89	93.37
**participant 5**	81.08	84.62	93.74	94.39
**participant 6**	89.86	90.12	89.83	91.47
**participant 7**	85.13	91.31	88.79	90.01
**participant 8**	86.87	85.98	92.85	93.95
**participant 9**	87.38	91.54	90.68	93.17
**participant 10**	82.78	83.65	94.24	94.25
**Average**	**83.71**	**86.98**	**92.72**	**93.83**
